# Response to hemolysis and DAT positivity

**DOI:** 10.1038/s41372-025-02308-y

**Published:** 2025-05-05

**Authors:** Shanice Wells, Ramya Balasubramanian, Khang Nguyen, David L. Schutzman

**Affiliations:** 1 Department of Pediatrics, Einstein Medical Center Philadelphia, Philadelphia, PA USA; 2 Division of Newborn Medicine, Maria Fareri Children’s Hospital, Westchester Medical Center, Valhalla, NY USA

**Keywords:** Metabolism, Diagnostic markers

We appreciate the commentary of doctors Kaplan and Hammerman on our paper “End-tidal carbon monoxide for routine monitoring of significant hemolysis in the management of newborn hyperbilirubinemia”. In particular, we appreciate, and agree with, their concern over the need to identify newborns with G6PD deficiency, in order to appropriately manage any hyperbilirubinemia they may experience. Although not specifically reported in the paper, of the 45 infants who were G6PD positive, only one showed evidence of significant hemolysis (an end-tidal carbon monoxide (ETCO) level ≥2.5 ppm).

While G6PD testing is part of the mandated newborn screening in Pennsylvania, as we noted in our paper, this result is not available until well after the infant has been discharged from the hospital, and therefore it was not available for our management of these infants. Interestingly, the current AAP guidelines make no mention of attempting to determine the etiology of significant hemolysis in the newborn other than from A/B/O or Rh incompatibility during the infant’s birth hospitalization [1]. We completely agree with doctors Kaplan and Hammerman’s conclusion stated 10 years ago that the optimal way to reduce kernicterus associated with G6PD deficiency would be routine point of care screening and parental education [2]. Unfortunately, 10 years later, point of care G6PD testing is still not commonly available.
